# Quasi-Drugs Developed in Japan for the Prevention or Treatment of Hyperpigmentary Disorders

**DOI:** 10.3390/ijms11062566

**Published:** 2010-06-18

**Authors:** Hideya Ando, Mary S. Matsui, Masamitsu Ichihashi

**Affiliations:** 1 Skin Aging and Photo-aging Research Center, Doshisha University, Kizugawa, Kyoto 619-0225, Japan; E-Mail: mm_ichihashi@hotmail.com; 2 Kobe Skin Research Institute, Kobe, Hyogo 650-0047, Japan; 3 Biological Research Division, The Estee Lauder Companies Inc., Melville, New York 11747, NY, USA; E-Mail: mmatsui@Estee.com

**Keywords:** melanin, melanocyte, pigmentation, skin

## Abstract

Excess production of melanin or its abnormal distribution, or both, can cause irregular hyperpigmentation of the skin, leading to melasma and age spots. To date, various quasi-drugs that prevent or improve hyperpigmentary disorders have been developed and officially approved by the Ministry of Health, Labor and Welfare of Japan. Many of these inhibit the activity of tyrosinase, an enzyme required for melanin synthesis, for example, by competitive or non-competitive inhibition of its catalytic activity, by inhibiting its maturation, or by accelerating its degradation. In this review, we categorize the quasi-drugs developed in Japan to prevent or treat hyperpigmentary disorders, or both, and discuss perspectives for future development.

## 1. Introduction

Skin lightening or whitening quasi-drugs (QDs), a category created in Japan for functional cosmetics, contain active ingredients that prevent or improve hyperpigmentation in disorders, such as melasma and solar lentigo. To be sold in Japan, they must be approved by the Japanese Ministry of Health, Labor and Welfare (MHLW). Although much still remains unknown about the causes of hyperpigmentation, it is thought that the localized and irregular over-production of melanin by melanocytes in the basal layer of the epidermis or the retention of melanin in the epidermis due to aberrant epidermal turnover, or both, play a role in hyperpigmentary disorders. Development of topical materials to treat these cosmetic hyperpigmentary disorders, with the goal of reducing excess melanin production and promoting clearing of accumulated melanin from the epidermis, has contributed significantly to the understanding of melanogenesis and pigmentation as physiological processes.

## 2. Instances of Skin Lightening QDs Developed in Japan

Until the late 1980s, ascorbic acid (also termed Vitamin C) and placental extracts were used as skin lightening QDs in Japan. Since 1988, starting with the approval of kojic acid by the MHLW, many companies have begun to develop proprietary QDs.

### 2.1. Ascorbic Acid and Its Derivatives

Melanin synthesis is regulated by the rate-limiting enzyme, tyrosinase, a membrane-bound copper-containing glycoprotein, which initiates the biosynthetic pathway of melanin by catalyzing the hydroxylation of tyrosine to DOPA. Since subsequent reactions in the melanin synthetic pathway, e.g., the conversion of DOPA to DOPAquinone, as well as other non-enzymatic reactions, are oxidative reactions, antioxidants such as ascorbic acid, are effective inhibitors of melanin synthesis. Ascorbic acid and its derivatives are the most popular skin lightening QDs that have ever been used in Japan.

Examples of ascorbic acid derivatives are magnesium *L*-ascorbic acid 2-phosphate (QD approval was obtained by the Takeda Pharmaceutical Company Limited in 1988), sodium L-ascorbic acid 2-phosphate, L-ascorbic acid 2-glucoside (obtained by Shiseido Co., Ltd. and Kaminomoto Co., Ltd. in 1994), and L-ascorbic acid ethyl ester (obtained by Shiseido Co., Ltd. in 2005). Although the inhibitory mechanism of skin lightening by L-ascorbic acid ethyl ester is to prevent polymerization of melanin monomers involved in the immediate pigment darkening of the skin induced by ultraviolet light A (UVA) (320–400 nm) [1,2], the inhibitory mechanisms of melanin synthesis by ascorbic acid and its other derivatives are mainly antioxidative in nature. L-Ascorbic acid 2-glucoside has been shown to maintain prolonged biological activity of ascorbic acid longer than sodium L-ascorbic acid 2-phosphate, a conventional ascorbic acid [3]. In clinical trials, a 10% magnesium L-ascorbic acid 2-phosphate-containing formulation was shown to be effective for reducing melasma and age spots [4]. A 2% L-ascorbic acid 2-glucoside-containing cream was shown to accelerate the disappearance of ultraviolet light B (UVB) (280–320 nm)—induced hyperpigmentation of the skin [5].

### 2.2. Placental Extracts

Placental extracts have long been used as an active ingredient for skin lightening QDs, together with ascorbic acid and its derivatives. Previously, a bovine-derived placental extract was the primary commercial source. However, swine-derived placental extracts are now used because of concern over mad cow disease. Various amino acids and minerals are at high concentrations in placental extracts, and the efficient inhibition of melanin synthesis and the enhancement of melanin removal from the skin, due to increased epidermal turnover have been reported [6,7]. Interestingly, although placental extract has a strong history of use to decrease hyperpigmentation, it has also been shown to increase melanogenesis. The lipid fraction of human placental extract was reported to increase melanin synthesis via the enhancement of tyrosinase gene expression [8].

### 2.3. Kojic Acid (Obtained by Sansho Seiyaku Co., Ltd. in 1988)

Kojic acid, a pyrone derivative obtained from the fermentation process of Japanese liquor, is known to have an antibacterial activity. Kojic acid was shown to inhibit the activity of tyrosinase by chelating copper atoms in its active site [9]. In clinical trials, a 1% kojic acid-containing formulation was shown to be effective for treating hyperpigmentary disorders, such as melasma, post-inflammatory hyperpigmentation, age spots, and freckles [10].

The MHLW in March, 2003 notified suppliers of kojic acid to delay manufacture or import because of caution about possible carcinogenic effects [11]. However, after revaluation in November, 2005, kojic acid is now accepted to be safe as a cosmetic ingredient, and continues to be used as a skin lightening QD [12].

### 2.4. Arbutin (Obtained by Shiseido Co., Ltd. in 1989)

Arbutin, a naturally occurring β-*D*-glucopyranoside derivative of hydroquinone, is found in cowberry leaves, and inhibits tyrosinase activity competitively, but at non-cytotoxic concentrations in cultured melanocytes [13]. A 3% arbutin-containing formulation was shown to be effective for treating hyperpigmentary disorders, such as melasma [14].

### 2.5. Ellagic Acid (Obtained by the Lion Corporation in 1996)

Ellagic acid is a naturally occurring polyphenol found in a variety of plants, such as strawberries, geraniums, and green tea. The inhibitory effect of ellagic acid on melanin synthesis is similar to kojic acid, *i.e.*, ellagic acid inhibits tyrosinase activity by chelating copper atoms in its active site [15]. A 0.5% ellagic acid-containing cream was shown to be effective for treating UVB-induced hyperpigmentation of the skin [16].

### 2.6. Chamomilla Extract (Obtained by the Kao Corporation in 1998)

Chamomilla extract has been used as a traditional anti-inflammatory agent, and is the only one so far, in Japan, that has been approved as a skin lightening QD from botanical extracts. It has been shown that keratinocytes secrete endothelin-1, a type of inflammatory cytokine, which activates melanocytes when UV is irradiated on the epidermis [17]. Chamomilla extracts have been shown to act as an antagonist for endothelin-receptor binding, which mediates cell-to-cell signaling between keratinocytes and melanocytes. This antagonism leads to the inhibition of melanin synthesis in melanocytes [18]. Until then, all earlier skin lightening QDs had been developed to inhibit tyrosinase activity. In contrast, the chamomilla extract is a unique QD focused on affecting the keratinocytes that surround melanocytes. A 0.5% chamomilla extract-containing cream was shown to be effective for treating UVB-induced hyperpigmentation of the skin [19,20].

### 2.7. 4-n-Butylresorcinol (Rucinol^®^) (Obtained by POLA in 1998)

Rucinol^®^ was selected by screening synthetic resorcinol derivatives that can elicit strong competitive inhibition of tyrosinase activity. Melanin synthesis is catalyzed by tyrosinase, together with tyrosinase-related proteins (TRP) −1 and −2, and Rucinol^®^ has been shown to inhibit melanin synthesis in cultured mouse melanocytes via direct inhibition not only of tyrosinase activity [21], but also of TRP-1 activity [22]. A 0.3% Rucinol^®^-containing lotion was shown to be effective for treating hyperpigmentary disorders, such as melasma [22].

### 2.8. Linoleic Acid (Obtained by Sunstar Inc. in 2001)

Linoleic acid is an unsaturated fatty acid (C18:2) derived from hydrolyzed botanical oils, such as safflower, and is a major component of biological cell membranes. Tyrosinase is degraded endogenously in melanocytes [23], and linoleic acid has been shown to accelerate tyrosinase degradation, and to decrease tyrosinase levels. These actions lead to the down-regulation of melanin synthesis [24]. In clinical trials, topical application of a 0.1% linoleic acid-containing liposomal formulation has been shown to be effective for treating melasma [25] and to lighten UVB-induced hyperpigmentation of the skin [26,27].

### 2.9. Tranexamic Acid (Obtained by Shiseido Co., Ltd. in 2002)

Tranexamic acid has been used as a traditional hemostatic medicine, and is known as an oral medicine for treating melasma [28]. Plasmin, a kind of protease in the blood serum, functions to enhance the intracellular release of arachidonic acid, a precursor of prostanoid [29], and also to elevate alpha-melanocyte stimulating hormone processed from pro-opiomelanocortin [30]. Both arachidonic acid and alpha-melanocyte stimulating hormone can activate melanin synthesis by melanocytes. Therefore, the anti-plasmin activity of tranexamic acid is thought to play a role in its topical effectiveness for treating melasma.

### 2.10. 4-Methoxy Potassium Salicylate (4MSK) (Obtained by Shiseido Co., Ltd. in 2003)

The inhibitory mechanism of 4MSK on melanin synthesis was shown to be the competitive inhibition of tyrosinase activity. This mechanism is similar to those of arbutin and Rucinol^®^.

### 2.11. Adenosine Monophosphate Disodium Salt (Obtained by Otsuka Pharmaceutical Co., Ltd. in 2004)

Adenosine monophosphate has the potency to increase the amount of intracellular glucose uptake, which is necessary for the biosynthesis of adenosine triphosphate, a source of intracellular energy. Therefore, adenosine monophosphate disodium salt accelerates epidermal turnover due to the elevated intracellular energy metabolism, which leads to the excretion of melanin from the skin, *i.e.*, adenosine monophosphate prevents the accumulation of melanin in the skin [31]. A 3% adenosine monophosphate disodium salt-containing formulation was shown to be effective for treating hyperpigmentary disorders, such as melasma [31].

### 2.12. 5,5′-Dipropyl-biphenyl-2,2′-diol (Magnolignan^®^) (Obtained by Kanebo Cosmetics Inc. in 2005)

Magnolignan^®^ is a biphenyl compound and a kind of polyphenol that has a structure similar to the magnolol and honokiol of Magnolia obovata. Tyrosinase is known to mature due to its glycosylation in the endoplasmic reticulum (ER) and Golgi apparatus; Magnolignan^®^ inhibits the maturation of tyrosinase, which leads to decreased melanin synthesis [32]. A 0.5% Magnolignan^®^-containing formulation was shown to be effective for treating UVB-induced hyperpigmentation of the skin [33] and is also effective in treating hyperpigmentary disorders, such as melasma and senile lentigo [34].

### 2.13. 4-(4-Hydroxyphenyl)-2-butanol (4-HPB) (Obtained by Kanebo Cosmetics Inc. in 2007)

4-HPB is a phenol compound found in extracts of White Birch and Nikko Maple. The inhibitory mechanism of 4-HPB on melanin synthesis was shown to be due to its competitive inhibition of tyrosinase activity.

### 2.14. Tranexamic Acid Cetyl Ester Hydrochloride (Obtained by CHANEL .KK in 2009)

The effect of tranexamic acid cetyl ester hydrochloride in treating hyperpigmentary disorders is due to the inhibition of UVB-induced inflammation, which leads to the quiescence of active melanocytes. This mechanism is similar to those of chamomilla extract and tranexamic acid.

## 3. Classification of the Mechanisms of QDs

To date, many private company-originated QDs have been developed that are based on various mechanisms of skin lightening or whitening efficacy (Table 1). The major target of those QDs is tyrosinase in melanocytes, while some QDs work on keratinocytes or on epidermal metabolism. Among the melanocyte-targeted QDs, the inhibitory mechanism targeting tyrosinase activity can be divided into two groups, *i.e.*, the inhibition of tyrosinase catalytic activity, such as anti-oxidation, the chelation of copper atoms in tyrosinase active site, and competitive inhibition, while the other category is the decrease of tyrosinase protein levels, which also leads to the inhibition of tyrosinase activity, such as accelerating tyrosinase degradation or inhibiting tyrosinase maturation, which eventually forwards immature tyrosinase to the ER-associated protein degradation pathway [35]. An efficient way to decrease tyrosinase protein levels is to reduce tyrosinase mRNA levels, as already reported for lipoic acid [36] and sphingosine-1-phosphate [37]. However, compounds that regulate transcription have not yet been approved by the MHLW of Japan for skin lightening QDs.

On the other hand, the keratinocyte-targeted QDs inhibit the activation of melanocytes by blocking or reducing UVB-induced inflammatory cytokines, and melanocyte quiescence eventually leads to decreased tyrosinase activity. In addition, the epidermis-targeted QDs elicit excretion of melanin from the epidermis, which leads to recovery from hyperpigmentary disorders. This is a special skin lightening strategy that is substantially independent from melanocyte function.

## 4. Perspectives

Nowadays, many of the hyperpigmentary disorders of the skin can be remedied by various laser therapies. Moreover, the chemical peeling of the stratum corneum using α-hydroxy acids, such as glycolic acid [38] and lactic acid [39], has also been shown to be modestly effective for treating hyperpigmentary disorders, such as melasma, due to the desquamation of melanin from the epidermis. In addition, this accelerated desquamation is often used to enhance the efficacy of other treatments.

Hydroquinone is currently the gold standard topical drug for dyschromia. It remains somewhat controversial however, and at one time, was available only by prescription, because it has been associated with several adverse events. For example, vitiligo-like effects and exogenous ochronosis have been reported after hydroquinone use [40]. In addition, the US FDA has stated that hydroquinone cannot be ruled out as a potential carcinogen and therefore is prohibited for use in cosmetics. However, a change of Japanese Pharmaceutical Law in 2001 now allows the use of hydroquinone as an ingredient of cosmetic formulations at lower and presumably safer concentrations. Hyperpigmentary conditions indicated for skin lightening QDs developed in Japan are limited, that is, clinical trials have demonstrated their efficacy mostly against melasma and solar lentigo. Nevertheless, those QDs remain popular, especially in Asian cosmetic markets, as a convenient means to treat non-pathological hyperpigmentary disorders of the skin.

To date, various skin lightening QDs have been developed based on a variety of functional mechanisms. When it comes to the investigation of skin lightening or whitening agents at a global scale, a large number of melanogenesis inhibitors, such as inhibitors of tyrosinase mRNA transcription, tyrosinase glycosylation, and tyrosinase catalytic activity, and accelerators of tyrosinase degradation have been reported [35,41–45]. Those findings will be further expanded and utilized for developing different strategies from previous theories for QDs, for instance, the inhibition of melanosome transfer from melanocytes to keratinocytes. Examples of inhibitors of melanosome transfer include soybean trypsin inhibitor [46], niacinamide [47], and centaureidin [48]. Since various types of hyperpigmentary disorders are based on different mechanisms, multi-functional topical formulations that combine existing skin lightening QDs, including not only inhibition of melanin synthesis, but also inhibition of inflammation or acceleration of epidermal turnover, or both, may be better strategies for increasing the efficacy of products to treat hyperpigmentary disorders, such as melasma and solar lentigo.

## Figures and Tables

**Table 1.** Mechanistic classification of skin lightening QDs approved by the MHLW of Japan.

***Target***	***Mechanism***		***Detail***	***Skin Lightening QD***	
**Melanocyte**	Inhibition of tyrosinase activity		Anti-oxidation	Ascorbic acid/derivatives	
			Chelating copper atoms	Kojic acid	Ellagic acid
			Competitive inhibition	Arbutin	Rucinol®
				4MSK	4-HPB
		Decrease of tyrosinase protein level	Acceleration of Tyr degradation	Linoleic acid	
			Inhibition of Tyr maturation	Magnolignan®	
**Keratinocyte**	Inhibition of KC-MC signaling		Inhibition of UV inflammation	Chamomilla extract Tranexamic acid/derivative	
**Epidermis**	Acceleration of epidermal turnover		Desquamation of melanin	Placental extract Adenosine mono-phosphate	

KC: keratinocyte, MC: melanocyte, Tyr: tyrosinase, UV: ultraviolet light
